# Subclinical and Borderline Rejection are Associated With Death‐Censored Graft Loss After Kidney Transplantation: A Systematic Review and Meta‐Analysis

**DOI:** 10.1111/ctr.70536

**Published:** 2026-04-08

**Authors:** Takayuki Yamada, Shota Obata, Arjun Kalaria, Abiha Abdullah, Bryce Parrish, Massiel Cruz Peralta, Vrishketan Sethi, Charbel Elias, Jason Mial‐Anthony, Michele Klein‐Fedyshin, Chethan Puttarajappa, Rajil Mehta, Aravind Cherukuri, Berkay Demirors, Michele Molinari

**Affiliations:** ^1^ Division of Nephrology, Omihachiman Community Medical Center Omihachiman Shiga Japan; ^2^ Department of Medicine, Division of Nephrology Stanford University School of Medicine Palo Alto USA; ^3^ Florida Kidney Physicians Jacksonville Florida USA; ^4^ University of Pittsburgh Pittsburgh USA; ^5^ Division of Transplant Nephrology, Thomas E. Starzl Transplantation Institute University of Pittsburgh Medical Center Pittsburgh USA; ^6^ Department of Surgery University of Pittsburgh Medical Center Pittsburgh USA; ^7^ Department of Surgery Division of Transplantation University of Pittsburgh Medical Center Pittsburgh USA; ^8^ University of Pittsburgh, Health Sciences Library System (MKF) Pittsburgh USA

**Keywords:** acute rejection, death‐censored graft loss, kidney transplant, rejection, subclinical inflammation, subclinical rejection

## Abstract

**Background:**

The prognostic significance of subclinical rejection (SCR) and borderline rejection (BLR) detected on surveillance kidney allograft biopsies remains uncertain.

**Methods:**

We performed a systematic review and meta‐analysis of studies enrolling adult kidney transplant (KT) recipients undergoing protocol biopsies. The primary outcome was death‐censored graft loss (DCGL); the secondary outcome was subsequent rejection. Random‐effects models, sensitivity analyses, and meta‐regression were used.

**Results:**

Fifteen studies (5428 recipients) were included. SCR was associated with a higher risk of DCGL (RR 2.22; 95% CI 1.67–2.95) and subsequent rejection (RR 3.09; 95% CI 2.34–4.09), with low heterogeneity. Both T cell–mediated rejection (RR 1.82; 95% CI 1.28–2.61) and antibody‐mediated rejection (ABMR) (RR 3.35; 95% CI 2.11–5.33) were associated with increased DCGL. BLR was associated with increased DCGL (RR 2.40; 95% CI 1.67–3.46) and subsequent rejection (RR 2.79; 95% CI 2.10–3.69). Findings were robust across sensitivity analyses, including contemporary Banff‐era definitions.

**Conclusions:**

SCR and BLR are associated with inferior long‐term graft outcomes. These findings underscore their prognostic significance and warrant prospective studies to determine optimal management strategies.

**Abbreviations**
Subclinical rejection: SCR
T‐cell mediated rejection: TCMR
Antibody mediated rejection: ABMR
Borderline rejection: BLR
Death‐censored graft loss: DCGL
Risk ratios: RRs
Hazard ratios: HRs
Confidence Interval: CI
Interstitial fibrosis and tubular atrophy: IFTA
Kidney Transplantation: KT
End‐Stage Kidney Disease: ESKD

## 1 Introduction

Kidney transplant (KT) is the preferred treatment for end‐stage kidney disease (ESKD), conferring improved survival and quality of life compared to dialysis [1]. With contemporary immunosuppression, one year allograft survival exceeds 95% [1, 2] However, despite marked reductions in overt acute rejection, gains in long‐term graft survival have been modest [3]. For instance, the 10‐year overall graft survival after deceased‐donor KT increased from 42.3% in 1996–1999 to only 53.6% in 2008–2011 [4].

Allograft failure reflects heterogeneous mechanisms, including acute or chronic T cell‐mediated rejection (TCMR), antibody‐mediated rejection (ABMR), calcineurin inhibitor toxicity, BK virus nephropathy, and interstitial fibrosis and tubular atrophy (IFTA) of uncertain etiology [5]. IFTA is commonly identified on late biopsies and may reflect cumulative injury, including subclinical alloimmune inflammation [6, 7].

Subclinical rejection (SCR), first described in 1990s, is defined as histologic rejection in the absence of clinical dysfunction, (e.g., stable serum creatinine and now new proteinuria) [8]. As a result, SCR is detected primarily through surveillance (protocol) biopsies. Reported incidence within the first posttransplant year ranges from 2.6% to 25% [9, 10, 11].

While several studies suggest that SCR contributes to progressive injury and fibrosis [12], its association with definitive outcomes such as subsequent rejection and death‐censored graft loss (DCGL) remains debated, with inconsistent findings across cohorts [13, 14, 15]. The clinical significance of borderline rejection (BLR), a histologic inflammation insufficient to meet full Banff criteria for acute rejection, also remains controversial [16].

To address these uncertainties, we conducted a systematic review and meta‐analysis to quantify associations between SCR, including histologic phenotypes such as TCMR and ABMR, and long‐term KT outcomes, focusing on DCGL and subsequent rejection.

## 2 Methods

### 2.1 Literature Search

This systematic review and meta‐analysis followed PRISMA 2020 and Cochrane Handbook for Systematic Reviews of Interventions [17, 18]. The protocol was registered in the International Prospective Register of Systematic Reviews (PROSPERO; CRD4202343563) and published previously [19].

We searched MEDLINE (PubMed), EMBASE (Elsevier), and Cochrane Central (Wiley) for studies published from January 1995 to January 2025. A trained health sciences librarian developed and executed search strategies (Table S1). We included both Medical Subject Headings (MeSH) and free‐text terms using Boolean logic and limited the results to English‐language articles to support consistent data extraction and risk‐of‐bias assessment. The search strategy was tested and validated against a known set of relevant articles. The MEDLINE search was translated to the syntax of the other databases. Grey literature was searched via Preprints in PubMed and EMBASE, conference abstracts in EMBASE and trials in Cochrane Central. After downloading the search results to EndNote (Clarivate), clear duplicates were removed using the process described by Bramer et al. [20]. In addition, the bibliographies of included studies and retrieved review articles were examined to identify additional relevant articles not found through database searches.

### 2.2 Study Selection

Eligible studies were prospective or retrospective observational studies and randomized controlled trials published in peer‐reviewed journals. Eligible studies reported outcomes in adult KT recipients (aged >18 years) who underwent surveillance biopsies with histologic assessment for SCR/BLR and reported outcomes with 2 or more years of follow‐up. There were no restrictions based on geographic location or country income level.

We excluded reviews, case reports, non‐human studies, and publications restricted to for‐cause biopsies, studies with less than 2 years follow‐up and studies lacking sufficient data to derive effect estimates after author contact.

Screening and data extraction were performed independently by multiple reviewers (TY, SO, AK, MCP, BP, AR, RM, MM) using DistillerSR (Version 2.37; DistillerSR Inc., 2022; https://www.distillersr.com). Disagreements were resolved by consensus or adjudication through discussion by a third reviewer (RM).

### 2.3 Aims of the Study

a) ***The primary objective*** was to evaluate the association between SCR and BLR and DCGL.

b) ***The secondary objective*** was to assess the association between SCR and BLR with subsequent rejection (TCMR and/or ABMR, as defined by individual studies).

### 2.4 Definitions of SCR and BLR

SCR was defined according to the criteria applied in each individual study and generally corresponded to Banff‐defined T cell‐mediated rejection (typically ≥ Banff 1A) or ABMR identified on protocol biopsy in the setting of stable graft function.

BLR was defined based on study‐level Banff criteria and typically reflected inflammatory changes below the threshold for definite acute rejection (e.g., below i2t2).

Because diagnostic thresholds and Banff classifications evolved over time, study‐specific definitions were extracted, and differences across studies were addressed through prespecified sensitivity analyses.

### 2.5 Data Extraction and Quality Assessment

Data were extracted using a standardized form. Retraction Watch and PubMed were systematically queried to identify retractions or published corrections before data extraction. For cohort studies, the risk of bias was assessed using the Newcastle‐Ottawa Scale (NOS) [21], with studies scoring al lest 6 points to be considered of higher methodological quality.

### 2.6 Statistical Analysis

Meta‐analyses were conducted using the meta and metafor packages in R (version 1.1‐0; R Foundation for Statistical Computing, Vienna, Austria). Time‐to‐event outcomes preferentially used adjusted hazard ratios (HRs) with corresponding 95% confidence intervals (CIs) reported in the original articles. If adjusted HRs were not reported, they were estimated from Kaplan–Meier (K‐M) curves using Parmar's method [22]. When HRs could not be derived, risk ratios (RRs) were calculated based on event data.

Random‐effects models were used to estimate pooled risk estimates across studies. Heterogeneity was assessed using the *I* ^2^ statistic and its associated *p*‐value. Heterogeneity was classified as low, moderate, or high based on *I* ^2^ thresholds of 25%, 50%, and 75%, respectively.

Prespecified subgroup analyses included SCR phenotypes (TCMR vs ABMR). Because Banff criteria and definitions of BLR varied across studies and over time, era‐restricted sensitivity analyses were performed (TCMR: Banff 2017+; ABMR: Banff 2013+). For BLR, we conducted a sensitivity analysis restricted to studies applying the minimum Banff i1t1 threshold to ensure comparability of pooled estimates. Additional sensitivity analyses evaluated biopsy timing (<3 vs ≥3 months), inclusion/exclusion of BLR, and treatment of SCR/BLR.

Meta‐regression evaluated study‐level moderators including sample size, recipient demographics, ESKD etiology, living donor proportion, induction, and maintenance immunosuppression. Publication bias was assessed by funnel plot inspection, Begg–Mazumdar, and Egger tests.

## 3 Results

### 3.1 Literature Search and Included Studies

**FIGURE 1:**
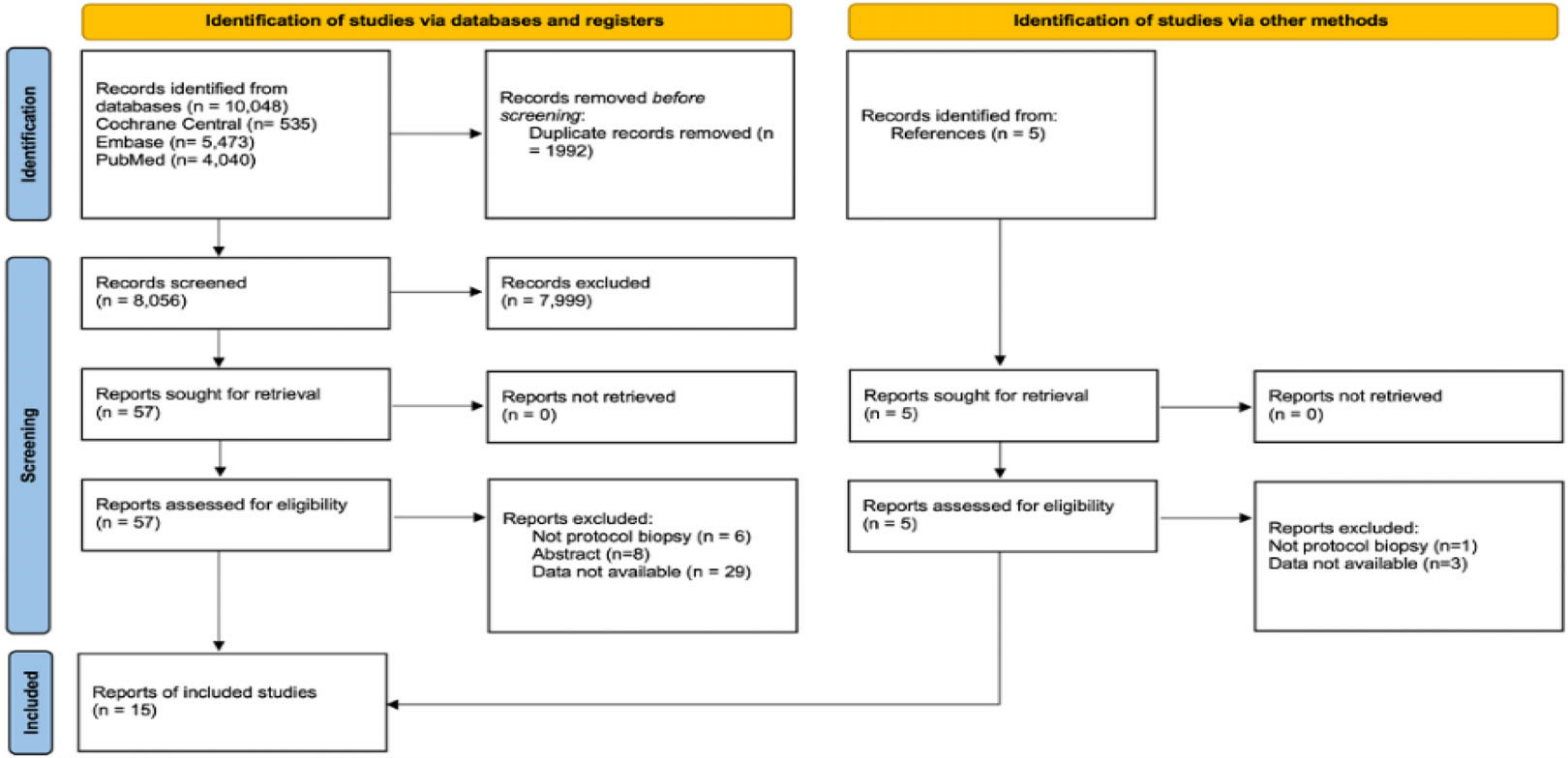
PRISMA flow diagram of study selection.

A diagram of the study selection is shown in Figure 1. A total of 10 048 records were identified through database searches (535 from Cochrane Central, 5473 from Embase, and 4040 from PubMed), and five studies were identified through reference screening. After removal of 1992 duplicates 8056 unique records were screened and 15 studies met the inclusion criteria [14, 23, 24, 25, 26, 27, 28, 29, 30, 31, 32, 33, 34, 35, 36]. These studies, published through January 2025, comprised 5428 adult KT recipients who underwent surveillance biopsies.

### 3.2 Study Characteristics and Quality Assessment

Study characteristics are summarized in Table 1. All studies were rated higher quality by NOS (≥6 stars; Table S2).

**TABLE 1:** Characteristics of Included Studies.

**Author**	**Country**	**Design**	**Banff criteria (Year)**	**N**	**Age, median**	**Male, %**	**Living donor, %**	**PB timing, months**	**SCR definition**	**SCR treatment**	**BLR treatment**	**HTN, %**	**DM, %**	**GN, %**	**PKD, %**	**HLA‐MM**	**ATG, %**	**BAS, %**	**FK, %**	**CsA, %**	**MMF, %**
Bertrand	France	RCS	2017	123	49.5	69.1	6.5	N/A	ABMR	Steroid or IVIG ± RTX ± PLEX	N/A	3.2	4.1	26.0	30.1	N/A	N/A	N/A	N/A	N/A	83.7
Choi	South Korea	RCS	1997	304	37.4	64.1	100.0	0.5	≥Banff 1A	Steroid	Steroid	37.3	38.5	40.2	N/A	2.8	N/A	N/A	9.9	90.1	18.1
Fernández‐Camargo	Mexico	RCS	2017, 2019	72	30.6	68.1	66.7	3, 12	≥Banff 1A	Steroid ± ATG	Steroid or no tx.	N/A	N/A	N/A	N/A	N/A	37.5	50.0	94.4	5.6	94.4
Gigliotti	Italy	RCS	1997	159	46.2	59.8	0.0	1	t≥1 and i>0	Steroid	N/A	N/A	N/A	N/A	N/A	N/A	0.0	100.0	64.9	35.1	100.0
Hoffman	US	RCS	2008	160	52.7	64.3	N/A	3, 12	≥Banff 1A	Steroid or ATG	N/A	N/A	N/A	N/A	N/A	N/A	97.0	0.0	100.0	0.0	100.0
Lee	Korea	PS‐matched cohort	2017	392	48.0	34.4	N/A	0.5, 12	≥Banff 1A	Steroid (TCMR); IVIG or PLEX (ABMR)	Steroid	10.2	28.7	36.4	3.5	N/A	56.0	44.0	100.0	0.0	100.0
Loupy	France	RCS	2011	1001	47.9	58.6	18.2	12	≥Banff 1A (TCMR); g>0 and/or ptc>0 (ABMR)	IVIG/PLEX + RTX; steroid; or no tx.	N/A	6.8	8.5	31.6	N/A	2.8	N/A	N/A	N/A	N/A	N/A
Mao	China	RCS	1997	227	40.3	61.7	0.0	1	≥Banff 1A	Steroid ± ALG, OKT3, or PLEX	No tx.	N/A	N/A	89.4	2.6	N/A	N/A	N/A	21.1	78.9	100.0
Mehta	US	PCS	2019	586	51.4	59.7	38.1	3, 12	≥Banff 1A	Steroid	No tx.	22.9	14.0	22.9	10.9	4.0	95.0	5.0	100.0	0.0	100.0
Nankivell	Australia	Cross‐sectional	1997	365	46.5	61.9	22.5	14.3	≥Banff 1A	Steroid pulse ± lymphocyte depletion	Steroid or no tx.	4.2	41.0	29.0	8.7	3.8	6.0	84.8	93.3	5.6	90.4
Ortiz	Finland	RCS	2011	516	48.5	64.0	1.8	3, 6, 12	Banff 1A and/or v≥0	N/A	N/A	N/A	14.8	30.5	14.0	N/A	11.2	11.3	27.5	61.6	70.0
Owoyemi	US	PCS	2017	205	51.5	63.4	44.4	3, 12	i2, t≥2 or v>0	Steroid ± ATG; PLEX/IVIG (ABMR)	N/A	20.0	18.0	3.9	9.3	N/A	94.1	N/A	N/A	N/A	N/A
Parajuli	US	RCS	2013	102	47.5	55.9	36.3	3, 12	ABMR	Steroid + PLEX/IVIG ± RTX	N/A	10.8	24.5	26.5	N/A	4.3	48.0	40.2	100.0	0.0	100.0
Rampersad	Canada	RCS	2019	775	49.0	N/A	48.0	1, 3, 6	Borderline	Steroid	Steroid	N/A	N/A	N/A	N/A	N/A	23.0	22.0	100.0	0.0	100.0
Seifart	US	RCS	2013–2019	441	49.0	61.9	39.9	6	i2t2 or v>0 (TCMR); g+ptc>1 (ABMR)	Steroid, ATG, IS increase, or no tx.	Same as SCR	N/A	24.5	33.3	N/A	N/A	68.5	11.1	98.4	0.2	97.5

Abbreviations: ABMR, antibody‐mediated rejection; ALG, anti‐lymphocyte globulin; ATG, anti‐thymocyte globulin; BAS, basiliximab; BLR, borderline rejection; CsA, cyclosporine; DM, diabetes mellitus; ESKD, end‐stage kidney disease; FK, tacrolimus; g, glomerulitis; GN, glomerulonephritis; HLA‐MM, human leukocyte antigen mismatch; HTN, hypertension; IS, immunosuppression; IVIG, intravenous immunoglobulin; MMF, mycophenolate mofetil; N/A, not available. Banff lesion scores: i, interstitial inflammation; PB, protocol biopsy; PCS, prospective cohort study; PKD, polycystic kidney disease; PLEX, plasmapheresis; PS, propensity score; ptc, peritubular capillaritis; RCS, retrospective cohort study; RTX, rituximab; SCR, subclinical rejection; t, tubulitis; TCMR, T‐cell–mediated rejection; tx., treatment; v, intimal arteritis.

### 3.3 Primary Outcomes

**FIGURE 2:**
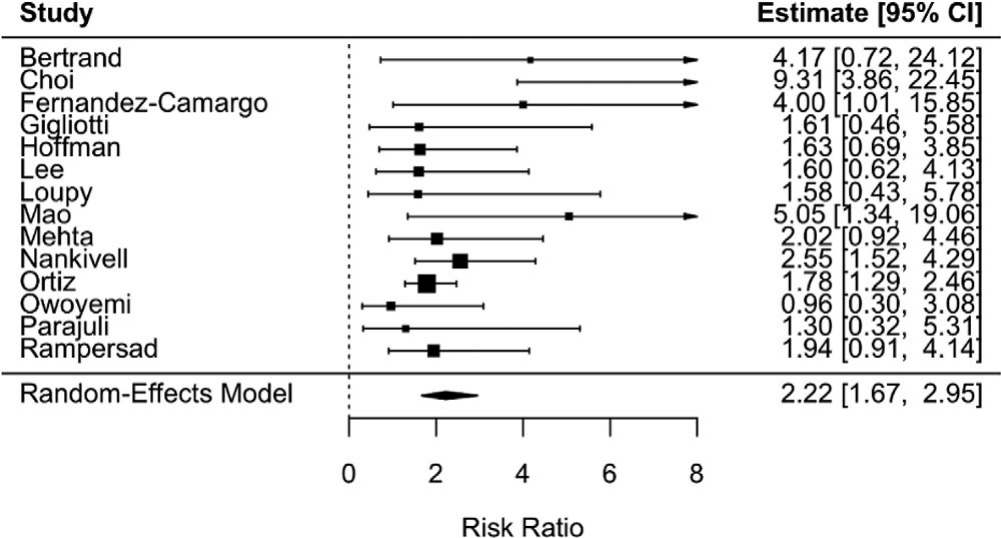
Association between SCR and DCGL. Forest plot of pooled RRs with 95% CIs.

**FIGURE 3:**
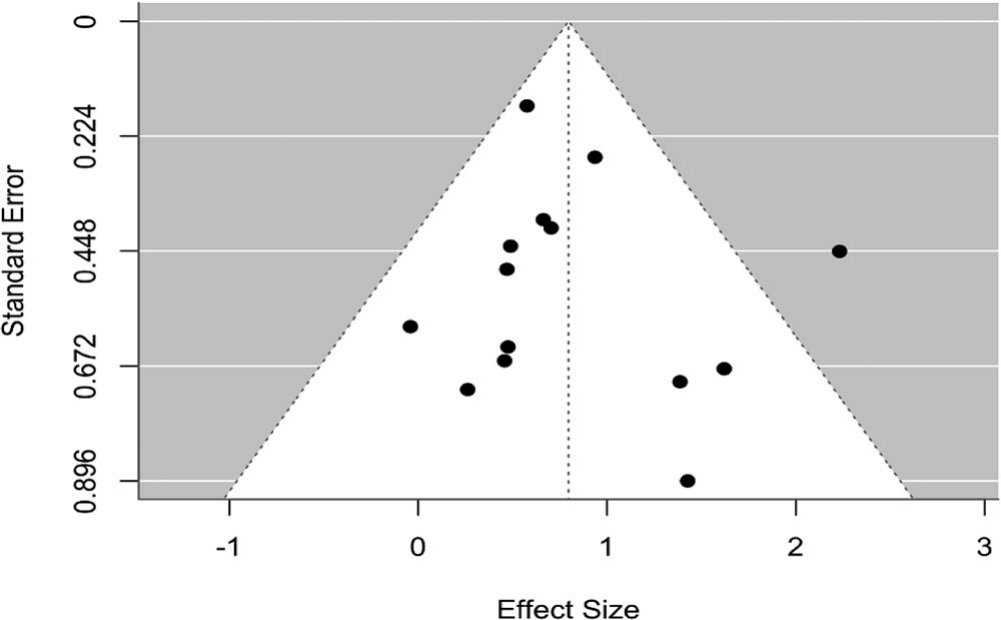
Funnel plot assessing potential publication bias for the association between rejection and DCGL. Legend: Each point represents an individual study plotted according to effect size (x‐axis) and corresponding standard error (y‐axis). The vertical dashed line indicates the pooled effect estimate from the random‐effects model. The diagonal dashed lines represent the 95% confidence limits around the pooled estimate. Symmetry of the plot suggests absence of small‐study effects or publication bias.

SCR was associated with increased risk of DCGL (RR 2.22; 95% CI 1.67–2.95), with low heterogeneity (*I* ^2^ = 32.1%) (Figure 2). There was no evidence of publication bias based on the Begg test (*p* = 0.74) or the Egger test (*p* = 0.69), and funnel plots demonstrated visual symmetry (Figure 3).

**FIGURE 4:**
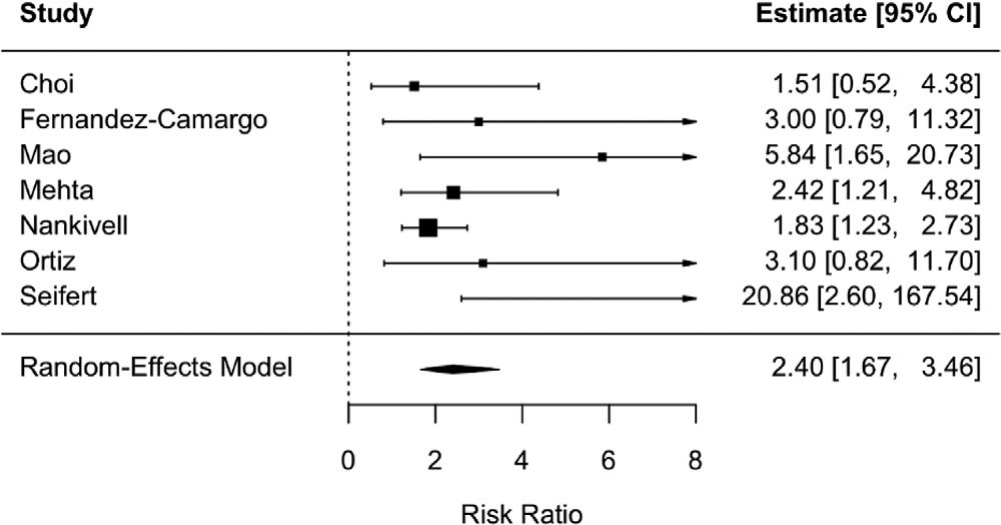
Association between BLR and DCGL. Forest plot of pooled RRs with 95% CIs.

BLR was also associated with an increased risk of DCGL (RR 2.40; 95% CI 1.67–3.46), with minimal heterogeneity across studies (*I* ^2^ = 15.0%) (Figure 4).

### 3.4 Secondary Outcomes

**FIGURE 5:**
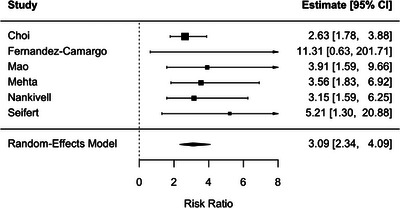
Association between SCR, defined according to study‐level Banff‐based criteria, and subsequent rejection. Forest plot of pooled RRs with 95% CIs.

**FIGURE 6:**
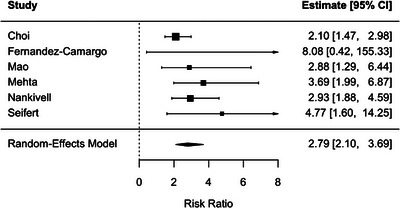
Association between BLR, defined according to study‐level Banff‐based criteria, and subsequent rejection. Forest plot reporting pooled RRs with 95% CIs.

SCR was associated with a significantly increased risk of subsequent rejection (RR 3.09, 95% CI: 2.34–4.09; *p* < .001) with no observer heterogeneity (*I* ^2^ = 0 %) (Figure 5). A similar association was observed for BLR (RR 2.79, 95% CI: 2.10–3.69; *P* < .001), with low among studies heterogeneity (*I* ^2^ = 18.0 %) (Figure 6).

### 3.5 Sensitivity Analyses

**FIGURE 7:**
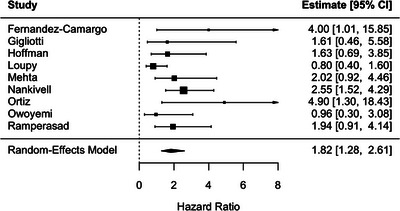
Association between T cell–mediated rejection and DCGL. Forest plot of pooled RRs with 95% CIs.

Sensitivity analyses consistently supported the robustness of the primary findings. In analyses restricted to 8 studies evaluating T cell‐mediated rejection (TCMR), TCMR was associated with an increased risk of DCGL (RR, 1.82; 95% CI, 1.28–2.61; *p* = 0.001), with low heterogeneity (*I* ^2^ = 32.5%) (Figure 7).

**FIGURE 8:**
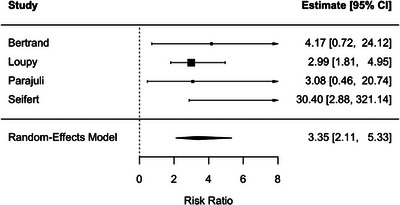
Association between ABMR and DCGL. Forest plot of pooled RRs with 95% CIs.

ABMR was associated with a higher risk of DCGL (RR, 3.35; 95% CI, 2.11–5.33; *p* < 0.001), with negligible heterogeneity (*I* ^2^ = 0.2%) (Figure 8).

**FIGURE 9:**
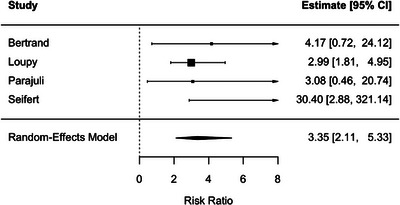
Association between T cell–mediated rejection defined according to Banff 2017 criteria or later and DCGL. Forest plot of pooled RRs with 95% CIs.

**FIGURE 10:**
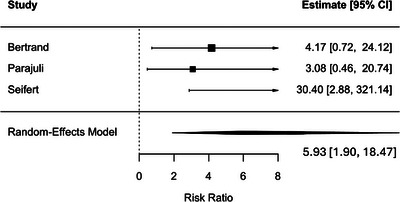
Association between ABMR defined according to Banff 2013 criteria or later and DCGL. Forest plot of pooled RRs with 95% CIs.

When analyses were limited to studies applying contemporary Banff criteria, TCMR defined according to Banff 2017 or later remained associated with an increased risk of DCGL (HR, 1.84; 95% CI, 1.22–2.78; *p* = 0.003), with no observed heterogeneity (*I* ^2^ = 0%) (Figure 9). Similarly, ABMR defined using Banff 2013 or later criteria was associated with a substantially increased risk of DCGL (RR, 5.93; 95% CI, 1.90–18.47; *p* = 0.002), with minimal heterogeneity (*I* ^2^ = 0.5%) (Figure 10).

**FIGURE 11:**
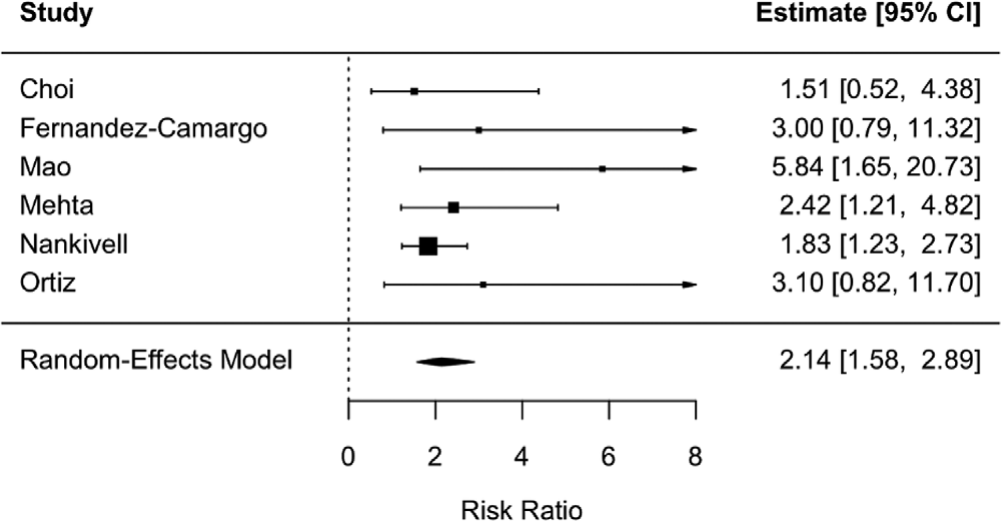
Association between BLR defined using the i1t1 threshold and DCGL. Forest plot of pooled RRs with 95% CIs.

**FIGURE 12:**
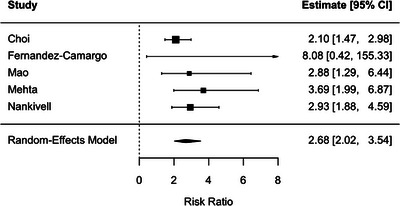
Association between BLR defined using the i1t1 threshold and subsequent rejection. Forest plot of pooled RRs with 95% CIs.

BLR, defined using the i1t1 threshold, was associated with higher risks of both DCGL (RR, 2.14; 95% CI, 1.58–2.89; *p* < 0.001; *I* ^2^ = 0%) and subsequent acute rejection (RR, 2.68; 95% CI, 2.02–3.54; *p* < 0.001; *I* ^2^ = 15.9%) (Figures 11 and 12).

The results of additional sensitivity analyses are summarized in Table 2. These were performed by:

1. exclusion of studies that classified BLR as SCR [26, 34]
2. exclusion of studies that did not report how SCR was treated [31],
3. restriction to studies in which protocol biopsies were performed at 3 months or later after transplantation [14, 25, 27, 29, 31, 32, 33]
4. restriction to studies reporting HRs only [24].

**TABLE 2:** Sensitivity analyses for DCGL (2A) and subsequent rejection (2B).

Sensitivity analysis	Effect type	Risk estimate	95% CI	*I* ^2^ (%)
**2A. Death‐censored graft loss**				
Excluding studies that classified BLR as SCR	RR	2.31	1.64–3.26	44.7
Excluding studies without SCR treatment details	RR	2.33	1.67–3.27	32.7
Only studies with protocol biopsies at ≥3 months	RR	1.78	1.39–2.27	0.0
Only studies reporting HRs	HR	1.94	1.58–2.39	0.0
**2B. Subsequent rejection**				
Defining BLR as minimum threshold i1t1	RR	2.14	1.58–2.89	0.0
Only studies with protocol biopsies at ≥3 months	RR	3.61	2.31–5.64	0.0

Abbreviations: BLR, borderline rejection; CI, confidence interval; HR, hazard ratio; *I* ^2^, heterogeneity statistic; RR, risk ratio; SCR, subclinical rejection.

Across all analyses, effect estimates remained consistent in direction and magnitude. In particular, HR‐based models (HR 1.94) and RR‐based models (RR 2.22) demonstrated clinically comparable effect sizes, with both approaches indicating approximately a two‐fold increased risk of DCGL, reinforcing the robustness of the primary findings.

### 3.6 Meta‐regression

Meta‐regression analysis of the association between SCR and DCGL identified living donor proportion (*p* = 0.009) and mycophenolate mofetil usage (*p* = 0.023) as statistically significant effect modifiers. Additionally, glomerulonephritis as a primary cause of ESKD demonstrated a trend toward explaining heterogeneity (*p* = 0.053) (Table 3).

**TABLE 3:** Meta‐regression analysis of predictors of DCGL.

Covariate	Coefficient	SE	95% CI	Z value	*p* value
CsA	0.0092	0.0051	−0.0007 to 0.0191	1.81	0.070
FK	−0.0079	0.0049	−0.0175 to 0.0017	−1.61	0.108
MMF	−0.0147	0.0064	−0.0273 to −0.0021	−2.28	0.023*
DM	0.0234	0.0154	−0.0068 to 0.0536	1.52	0.129
HTN	0.0308	0.0211	−0.0105 to 0.0721	1.46	0.144
GN	0.0210	0.0108	−0.0002 to 0.0422	1.94	0.053
PKD	−0.0074	0.0285	−0.0632 to 0.0485	−0.26	0.796
Living donor	0.0107	0.0041	0.0027 to 0.0188	2.62	0.009*

Abbreviations: CI, confidence interval; CsA, cyclosporine; DM, diabetes mellitus; FK, tacrolimus; GN, glomerulonephritis; HTN, hypertension; MMF, mycophenolate mofetil; PKD, polycystic kidney disease.

* indicates statistical significance (*p* < 0.05); SE, standard error.

## 4 Discussion

Before this study, the prognostic significance of SCR and BLR identified on surveillance biopsy remained uncertain. Prior reports were heterogeneous, clinical responses varied widely, and BLR was often regarded as a minor or inconclusive finding. In this systematic review and meta‐analysis, the data indicate that SCR, including TCMR and ABMR, is associated with a 2.0–3.0‐fold higher risk of DCGL and subsequent rejection in adult KT recipients. BLR was also associated with adverse outcomes, with an approximately 2.0‐fold higher risk of DCGL and a 2.7–2.8‐fold higher risk of subsequent rejection. These associations were consistent across sensitivity analyses and Banff eras, suggesting relevance to contemporary transplant practice [14, 25, 26, 29, 31, 32, 33, 34, 35, 36].

A key implication of these findings is that histologic inflammation carries prognostic information even when graft function appears stable. Posttransplant monitoring commonly relies on serum creatinine and proteinuria, yet these markers may remain unchanged despite ongoing alloimmune activity [8, 11]. The present results suggest that surveillance biopsy can identify clinically stable recipients who are nonetheless at increased long‐term risk, indicating that functional stability alone is an incomplete marker of immunologic risk.

BLR warrants specific consideration. SCR meets established Banff criteria for acute rejection and is generally treated, whereas BLR falls below diagnostic thresholds and is often managed conservatively. In this analysis, BLR was associated with risks of graft loss and subsequent rejection that were comparable in magnitude to those observed for SCR. These findings suggest that BLR reflects biologically relevant alloimmune activity rather than a benign or inconsequential process. The distinction between SCR and BLR therefore appears to reflect differences in severity rather than fundamentally different mechanisms, with prognosis driven by the presence and persistence of inflammation rather than by categorical labels [16, 25, 35, 36].

The observed associations are consistent with established mechanisms of chronic allograft injury. Inflammation within areas of tubular atrophy (i‐IFTA) is strongly associated with graft failure, and subclinical inflammation has been linked to progression of IFTA, providing a plausible pathway from early immune‐mediated injury to irreversible structural damage [6, 7, 12]. Phenotype‐specific mechanisms further support this interpretation. Persistent subclinical TCMR is associated with interstitial inflammation and tubulitis that may promote fibrotic remodeling, whereas ABMR is associated with DSA formation, endothelial injury, and microvascular inflammation, processes known to accelerate chronic graft injury even when graft function is preserved [16, 23, 33, 34, 36].

Immunologic risk at the donor–recipient level provides additional context. Molecular HLA mismatch, particularly at the eplet level, has been associated with DSA development, rejection, and graft loss [37, 38]. Within this framework, subclinical inflammation may represent the tissue‐level manifestation of sustained alloimmune pressure in higher‐risk donor–recipient pairs. BLR may reflect an earlier stage of this process, whereas SCR may represent a more advanced stage along the same biological continuum.

These findings have implications for posttransplant surveillance but do not define a single management strategy. Protocol biopsy practices vary widely across centers due to differences in institutional culture, perceived procedural risk, and resource availability [11, 15]. In addition, treatment following detection of SCR or BLR was heterogeneous and inconsistently reported across studies, limiting inference regarding the benefit of specific interventions. Accordingly, the results support the use of SCR and BLR as prognostic markers and argue for standardized, risk‐based response pathways, but they do not establish that routine treatment of all cases improves long‐term outcomes. Prospective studies are needed to define when intervention is beneficial, and which patients can be safely observed.

Noninvasive biomarkers may support risk stratification but are unlikely to replace histologic assessment. dd‐cfDNA correlates with acute rejection and DSA development, yet sensitivity for low‐grade TCMR and BLR remains limited, clinically actionable thresholds are uncertain, and cost‐effectiveness analyses have yielded mixed results [39, 40, 41, 42, 43]. A negative biomarker result should therefore not be interpreted as exclusion of histologic inflammation. In cases where SCR or BLR is detected on surveillance biopsy, histologic findings remain the primary determinant of risk assessment, and biomarkers may serve as complementary tools for longitudinal monitoring rather than definitive rule‐out tests.

Biopsy timing varied across included studies, ranging from early (<3 months) to later surveillance periods. Sensitivity analyses restricted to biopsies performed ≥3 months posttransplant yielded consistent effect estimates with low heterogeneity, suggesting that the observed associations were not driven by early peri‐transplant biopsies. Nevertheless, we cannot exclude the possibility that the prognostic weight of SCR or BLR differs between early and later detection, and timing‐specific effects warrant further investigation in prospective studies.

Meta‐regression analysis suggested that studies with a higher proportion of living donor transplants demonstrated a stronger association between SCR and DCGL. Data directly comparing the prognostic impact of SCR between living and deceased donor grafts are limited. One possible explanation is that living donor grafts experience minimal cold ischemia and lower rates of ischemia–reperfusion injury and delayed graft function, resulting in more favorable baseline outcomes. In this setting, SCR may represent a more direct contributor to graft injury, whereas in deceased donor grafts, early non‐immunologic injury may attenuate its relative impact. This observation remains hypothesis‐generating and warrants further study.

This study has several strengths and limitations that should be considered when interpreting the findings. The analysis includes more than 5400 KT recipients from multiple centers and eras and follows a registered protocol with librarian‐led searches and extensive sensitivity analyses, including restriction to contemporary Banff definitions. These features support the consistency and reproducibility of the results. At the same time, most included studies were observational and single center, with variation in biopsy timing, immunosuppression, and management of SCR and BLR, reflecting differences in routine clinical practice. Histological interpretation is inherently subjective, and lesion‐level analyses were not possible because of inconsistent reporting. Treatment effects could not be evaluated because post‐biopsy management was heterogeneous and incompletely documented. Although residual confounding cannot be excluded, the stability of results across sensitivity analyses and the generally low heterogeneity in key comparisons suggest that the observed associations are not driven by isolated studies or analytic choices.

In summary, the data indicate that SCR and BLR detected on surveillance biopsy are associated with worse long‐term KT outcomes. BLR should not be assumed to be a benign finding, and functional stability alone does not reliably indicate low immunologic risk. These findings underscore the prognostic relevance of subclinical inflammation and highlight the need for prospective trials to clarify optimal strategies for integrating histology, biomarkers, and molecular immunologic risk assessment in the pursuit of improved long‐term graft survival.

## Author Contributions

**Takayuki Yamada and Shota Obata:** methodology, data extraction, data analysis and writing and editing of protocol. **Arjun Kalaria:** methodology, data extraction, data analysis, and writing of protocol. **Massiel Cruz Peralta and Bryce Parrish:** data extraction and data analysis. **Abiha Abdullah, Vrishketan Sethi, Charbel Elias, and Jason Mial‐Anthony:** data extraction and editing of the protocol. **Michele Klein‐Fedyshin:** writing search terms, designing and conducting database searches, downloading and removing duplicates from the searches, writing search methodology for protocol and manuscript, editing manuscript and data extraction. **Chethan Puttarajappa, Aravind Cherukuri, Rajil Mehta, and Michele Molinari, Berkay Demirors:** conceptualization, methodology, data extraction, data analysis and editing of the protocol.

## Funding

This research received no specific grant from any funding agency in the public, commercial or not‐for‐profit sectors.

## Conflicts of Interest

The authors declare no conflicts of interest.

## Supporting information




**Supplementary File1**: ctr70536‐sup‐0001‐SuppMat.docx

## Data Availability

No new participant‐level data were generated for this study. All analyses are based on aggregate summary statistics reported in the cited publications. The data that supports the finding of the study (data‐extraction spreadsheet and R scripts) are available from the corresponding author on reasonable request.
