# Supplementary material for: Subclinical and Borderline Rejection are Associated With Death‐Censored Graft Loss After Kidney Transplantation: A Systematic Review and Meta‐Analysis
Source: Clin Transplant. 2026 Apr 8;40(4):e70536. doi: 10.1111/ctr.70536 (PMC13059068; doi:10.1111/ctr.70536)
Supplement: Supplementary file 1 — Supplementary File1: ctr70536‐sup‐0001‐SuppMat.docx [file CTR-40-e70536-s001.docx]

**Supplementary Tables and Figures**

**Supplementary Table 1A. PubMed (MEDLINE) Search Strategy**

| Step | Search Concept | Search Terms |
| --- | --- | --- |
| 1 | Subclinical rejection terms | ("Antibody-mediated rejection"[Title/Abstract] OR "Banff borderline"[Title/Abstract] OR "Banff score"[Title/Abstract] OR "borderline inflammation"[Title/Abstract] OR "borderline rejection"[Title/Abstract:~2] OR "cellular rejection"[Title/Abstract] OR "follow-up biopsies"[Title/Abstract] OR "follow-up biopsy"[Title/Abstract] OR "protocol biopsies"[Title/Abstract] OR "protocol biopsy"[Title/Abstract] OR "subclinical ABMR"[Title/Abstract] OR "subclinical inflammation"[Title/Abstract:~3] OR "subclinical rejection"[Title/Abstract:~3] OR "subclinical TCMR"[Title/Abstract] OR "surveillance biopsies"[Title/Abstract:~2] OR "surveillance biopsy"[Title/Abstract:~2] OR "T cell mediated rejection"[Title/Abstract]) |
| 2 | Biopsy AND subclinical terms | (("Biopsy"[MeSH Terms] OR "Biopsy"[Title/Abstract] OR "biopsies"[Title/Abstract] OR histolog*[Title/Abstract]) AND ("subclinical"[Title/Abstract] OR "Banff"[All Fields])) |
| 3 | Subclinical, kidney, transplant in title | ("subclinical"[Title] AND "kidney"[Title] AND transplant*[Title]) |
| 4 | Combined subclinical concepts | Step 1 OR Step 2 OR Step 3 |
| 5 | Kidney transplantation terms | ("Kidney Transplantation"[MeSH Terms] OR "Allograft nephropathy"[Title/Abstract] OR "kidney allograft*"[Title/Abstract] OR "kidney graft*"[Title/Abstract] OR "kidney recipient*"[Title/Abstract] OR "kidney transplant*"[Title/Abstract] OR "renal allograft*"[Title/Abstract] OR "renal transplant*"[Title/Abstract] OR "transplanted kidney"[Title/Abstract] OR ("Kidney"[MeSH Terms] AND "Allografts"[MeSH Terms])) |
| 6 | Graft outcome terms | ("Graft Rejection"[MeSH Terms] OR "Graft Survival"[MeSH Terms] OR "allograft dysfunction"[Title/Abstract] OR "allograft failure"[Title/Abstract] OR "allograft function"[Title/Abstract] OR "allograft histology"[Title/Abstract] OR "allograft histopathology"[Title/Abstract] OR "allograft inflammation"[Title/Abstract] OR "allograft loss"[Title/Abstract] OR "allograft nephropathy"[Title/Abstract] OR "allograft rejection"[Title/Abstract] OR "graft failure"[Title/Abstract] OR "graft function"[Title/Abstract] OR "graft loss*"[Title/Abstract] OR "graft outcome*"[Title/Abstract] OR "graft rejection"[Title/Abstract] OR "graft survival"[Title/Abstract] OR "histologic outcomes"[Title/Abstract] OR "immunological outcomes"[Title/Abstract] OR "renal function"[Title/Abstract] OR "transplant outcomes"[Title/Abstract] OR "transplant rejection"[Title/Abstract]) |
| 7 | Core search | Step 4 AND Step 5 AND Step 6 |
| 8 | Language limit | English[lang] |
| 9 | Human adults | ("Humans"[MeSH Terms] AND "Adult"[MeSH Terms]) |
| 10 | Apply limits | Step 7 AND Step 8 AND Step 9 |
| 11 | Exclusions (animals, pediatrics, publication types) | ("animals"[mh:noexp] OR "child"[MeSH Terms] OR "infant"[MeSH Terms] OR "pediatrics"[MeSH Terms] OR animal*[tiab] OR pediatric*[tiab] OR case reports[Publication Type] OR autobiography[Publication Type]) |
| 12 | Final PubMed set | Step 10 NOT Step 11 |
| 13 | Date limit | 1995/01/01–2024/12/31[pdat] |
| 14 | Final search | Step 12 AND Step 13 |

**Supplementary Table 1B. Embase Search Strategy**

| Step | Search Concept | Search Terms |
| --- | --- | --- |
| 1 | Rejection terms | 'antibody mediated rejection'/de OR 'banff classification'/exp OR 'banff score'/de OR 'biopsy technique'/de OR 'histopathology'/mj OR 'kidney biopsy'/de OR 'renal allograft biopsy'/exp OR 'subclinical rejection'/exp OR 't cell mediated rejection'/exp OR ('antibody-mediated rejection':ab,ti OR 'banff score':ab,ti OR 'borderline inflammation':ab,ti OR (borderline NEXT/2 rejection):ab,ti OR 'protocol biopsy':ab,ti OR 'subclinical abmr':ab,ti OR (subclinical NEXT/2 rejection):ab,ti OR 'subclinical tcmr':ab,ti OR (surveillance NEXT/2 biopsy):ab,ti) |
| 2 | Kidney transplantation terms | ('kidney transplantation'/exp OR kidney) AND ('allograft'/de OR 'renal graft dysfunction'/exp OR 'kidney allograft':ab,ti OR (kidney NEXT/1 transplant*):ab,ti OR (renal NEXT/1 allograft*):ab,ti) |
| 3 | Graft outcome terms | 'graft failure'/de OR 'graft rejection'/de OR 'graft survival'/de OR 'kidney function'/de OR 'allograft dysfunction':ab,ti OR 'allograft loss':ab,ti OR 'graft outcome*':ab,ti OR 'transplant outcomes':ab,ti |
| 4 | Core search | Step 1 AND Step 2 AND Step 3 |
| 5 | Title-focused subclinical search | (subclinical:ti AND (kidney:ti OR renal:ti) AND (transplant*:ti OR allograft*:ti)) |
| 6 | Apply limits | (Step 4 OR Step 5) AND [humans]/lim AND [english]/lim AND [1995–2024]/py |
| 7 | Exclusions | 'animal'/exp OR 'child'/exp OR 'infant'/exp OR 'pediatrics'/exp OR 'case report'/exp |
| 8 | Final Embase set | Step 6 NOT Step 7 |

**Supplementary Table 1C. Cochrane Library Search Strategy**

| Step | Search Concept | Search Terms |
| --- | --- | --- |
| 1 | Rejection terms | "Antibody-mediated rejection" OR "Banff borderline" OR "Banff score" OR "borderline inflammation" OR "borderline rejection" OR "follow-up biopsies" OR "protocol biopsies" OR "subclinical ABMR" OR "subclinical inflammation" OR "subclinical rejection" OR "subclinical TCMR" OR "surveillance biopsy" OR "T cell mediated rejection" |
| 2 | Kidney terms | kidney OR renal |
| 3 | Transplant terms | allograft* OR transplant* |
| 4 | Core search | Step 1 AND Step 2 AND Step 3 |
| 5 | Exclusions | animal* OR child* OR infant* OR pediatric* OR fetus OR fetal OR mouse OR mice OR rat OR rats |
| 6 | Final Cochrane set | Step 4 NOT Step 5 |
| 7 | Limits | English language; publication years 1995–2024 |

**Supplemental Table 2:**

Newcastle Ottawa Score for included studies

|  | Selection |  |  |  | Comparability | Outcome |  |  |  |
| --- | --- | --- | --- | --- | --- | --- | --- | --- | --- |
| Primary Author | Reperesentativeness of exposed cohort | Selection of non-exposed cohort | Ascertainment of exposure | Demonstration that outcome of interest was not present at start of study | Comparability of cohorts on basis of the design or analysis | Assessment of outcome | Was follow-up long enough for outcomes to occur | Adequay of follow up of cohorts | Total quality score |
|  |  |  |  |  |  |  |  |  |  |
| Author | n star | n. star | n stars | n stars | n stars | n stars | n stars | n stars | total n. stars |
| Bertrand | * | - | * | * | ** | * | * | * | 7 |
| Choi | - | - | * | * | ** | * | * | - | 7 |
| Fernández-Camargo | - | * | * | * | ** | * | * | * | 8 |
| Gigliotti | - | * | * | * | ** | * | * | - | 7 |
| Hoffman | - | * | * | * | ** | * | * | * | 8 |
| Lee | - | * | * | * | ** | * | * | * | 8 |
| Loupy | - | - | * | * | ** | * | * | * | 7 |
| Mao | - | * | * | * | * | * | * | - | 6 |
| Mehta | - | * | * | * | ** | * | * | * | 8 |
| Ortiz | - | * | * | * | ** | * | * | * | 8 |
| Owoyemi | - | * | * | * | ** | * | * | * | 8 |
| Parajuli | - | * | * | * | * | * | * | * | 7 |
| Rampersad | - | * | * | * | ** | * | * | * | 8 |
| Seifert | - | * | * | * | ** | * | - | * | 7 |
